# Correlates of sexually transmitted infections among Syrian refugee women and girls in Lebanon: knowledge, symptoms, and health-seeking behaviors

**DOI:** 10.1186/s12905-025-04036-z

**Published:** 2025-10-08

**Authors:** Dalia Sarieddine, Zahraa Chamseddine, Hady Naal, Asmaa El Dakdouki, Gladys Honein Abou Haidar, Hani Tamim, Tania Bosqui, Fouad Fouad, Sara Ibrahim, Zahi Abdul Sater, Shadi Saleh

**Affiliations:** 1 Global Health Institute, American University of Beirut, Beirut, Lebanon; 2 Hariri School of Nursing, American University of Beirut, Beirut, Lebanon; 3 Department of Internal Medicine, Clinical Research Institute, American University of Beirut, Beirut, Lebanon; 4 College of Medicine, Alfaisal University, Riyadh, Saudi Arabia; 5 Department of Psychology, American University of Beirut, Beirut, Lebanon; 6 Trinity Center for Global Health, Trinity College Dublin, Dublin, Ireland; 7 Department of International Public Health, Liverpool School of Tropical Medicine, Liverpool, UK; 8 Department of Health Policy and Management, American University of Beirut, Beirut, Lebanon; 9 Faculty of Health Sciences, American University of Beirut, Beirut, Lebanon

**Keywords:** Refugees; sexual reproductive health, HIV/AIDS, Lebanon, STI, Family planning

## Abstract

**Background:**

Syrian refugee girls and young women in Lebanon face a disproportionate risk of poor Sexual Reproductive Health (SRH) outcomes, especially Sexually Transmitted Infections (STIs). However, limited research has explored the factors associated with SRH vulnerabilities. This study aimed to quantify the associations between sociodemographic and clinical characteristics, and experiences of self-reported STI symptoms, health-seeking behaviors, and knowledge of HIV/AIDS.

**Methods:**

This study is part of the Self-Efficacy and Knowledge (SEEK) Trial, which aims to improve SRH and Family Planning (FP) among Syrian refugee women and girls in humanitarian settings. Baseline data (*n* = 485) were collected from two primary healthcare centers in the Bekaa in Lebanon, using the PAPFAM tool during November and December 2023.

**Results:**

Findings highlight some factors that align and others that contradict previous literature, as discussed in the manuscript. Logistic regression models showed that reporting STI symptoms was significantly associated with younger age of participants (aOR = 0.58, 95% CI=[0.25, 0.84], p-value = 0.025), financial barriers to seeking healthcare (aOR = 1.99, 95% CI = [1.07, 3.69], p-value = 0.028), and use of FP methods (aOR = 1.88, 95%CI= [1.01,3.51], p-value = 0.045). Better knowledge of HIV/AIDS was significantly associated with higher education of participants (aOR = 2.12, 95% CI = [1.08, 4.11], p-value = 0.028), higher age of spouse (aOR = 3.08, 95% CI= [1.97, 4.83], p-value < 0.001), and use of FP methods (aOR = 1.604, 95% CI=[1.01, 2.52], p-value = 0.042). Knowledge of HIV/AIDS transmission was also significantly associated with higher spouse age (aOR = 2.62, 95% CI=[1.46, 4.70], p-value = 0.001), higher education of participants (aOR = 3.99, 95% CI = [2.33, 5.64], p-value < 0.001), and use of FP methods (aOR = 3.21, 95% CI [1.63, 6.33], p-value = 0.001) .

**Conclusion:**

This study highlights the role of key factors associated with the experience of self-reported STI symptoms, knowledge of HIV/AIDS, and health-seeking behaviors. Findings suggest that age, education, economic barriers, and use ofFP methods should be considered in targeted interventions aiming to improve SRH outcomes among this population.

**Trial registration:**

Clinical Trial Number NCT07008950 initial release on February 28th 2025 and last release on June 5th 2025 with the clinical trial registry at National Institute of Health (NIH) protocol registration system.

**Supplementary Information:**

The online version contains supplementary material available at 10.1186/s12905-025-04036-z.

## Introduction

Sexually transmitted infections (STIs) remain a major public health challenge, with the World Health Organization (WHO) estimating over one millioncurable STIs acquired daily among individuals aged 15–49 years [1–3]. In 2020, approximately 374 million new infections were attributed to chlamydia, gonorrhea, syphilis, and trichomoniasis [3]. Beyond these, viral STIs such as herpes simplex virus type 2 (HSV-2) and human papillomavirus contribute substantially to morbidity, with human papillomavirus linked to over 311 cancer deaths annually and HSV-2 affecting 520 million people globally [3]. These STIs pose serious threats to Sexual and Reproductive Health (SRH), especially among women, leading to severe complications, including mother-to-child transmission, pelvicinflammatory disease, cervical cancer, and infertility [4].

Despite their significant impact on SRH, the urgency of addressing STIs and HIV/AIDS is often overlooked in humanitarian responses, particularly for marginalized populations like refugees [5]. This is especially true for adolescent girls and young women refugees, who make up a substantial majority [6, 7] and whose SRH needs remain largely unmet, increasing their vulnerability to HIV and other STIs [8]. Several displacement-related factors contribute to this heightened vulnerability, including unsanitary living conditions in refugee camps, insecurity, socioeconomic deprivation, sexual abuse, multiple partners, transactional sex, stigmatization, and limited access to preventive and educational resources [4, 5, 9, 10].

However, despite this elevated risk, recent studies on specific populations of Syrian refugees in Lebanon indicate a more nuanced picture regarding the actual burden of curable STIs. For instance, a recent study among pregnant Syrian refugee women seeking antenatal care (ANC) in Lebanon reported a low prevalence of chlamydia trachomatis (0.5%), neisseria gonorrhoeae (0%), and trichomonas vaginalis (0.2%). Although ANC clinics offer a practical entry point for reaching Syrian refugee women, with ANC engagement estimated to exceed 80% in Lebanon, these findings likely reflect a narrow subpopulation with regular access to maternal health services [14]. As such, they may not adequately represent the broader sexual and reproductive health (SRH) vulnerabilities faced by adolescent girls and young women, particularly those not engaged in ANC [14].

Furthermore, many Syrian refugee women do not seek SRH care due to a range of barriers, including financial constraints, transportation difficulties, lack of female healthcare providers, fear of mistreatment, security risks, poor SRH literacy, and lack of awareness of available SRH services [6, 12, 13]. Sociocultural barriers, religious norms, and pervasive stigma around STIs also play a major role in limiting refugee populations’ access to SRH knowledge and services, restricting awareness of STIs, and limiting open discussions on these issues [4, 15–19]. In particular, studies on Syrian refugee young women in Lebanon revealed significant gaps in STI knowledge, with 54.2% unable to identify any STIs and 20.5% unaware of associated symptoms [6]. These gaps are also largely attributed to reliance on informal sources of SRH information such as their peers, early marriage that disrupts access to SRH education, and stigma, all of which increase their vulnerability to SRH problems and reduce their overall health-seeking behaviors [10, 20–22].

Despite the growing recognition of SRH challenges faced by Syrian refugee women and girls in Lebanon, there remains a significant gap in the literature on factors influencing their experience of STIs, knowledge, and health-seeking behaviors [10, 23]. To our knowledge, this study is the first to examine the factorsassociated with self-reported STI symptoms, knowledge of STIs, particularly HIV/AIDS, and health-seeking behaviors for STIs among Syrian adolescent girls and young women refugees in Lebanon. By focusing on this rural and underserved population, our study provides novel insights that can inform targeted interventions and policies tailored to the unique needs of refugee adolescent girls and young women in similar contexts.

## Methodology

### SEEK trial

This study is conducted as part of the broader Self-Efficacy and Knowledge (SEEK) trial (protocol paper under review at the time of submission), which is a community-based Randomized Controlled Trial (RCT) that aims to enhance Family Planning (FP), SRH, and overall well-being in humanitarian settings. The trial involved developing, implementing, and evaluating the effectiveness of a WHO low-intensity/low-resource integrated intervention package on SRH, FP, and Mental Health (MH), specifically among Syrian adolescent girls and young women refugees aged 15 to 24 in the Bekaa valley, Lebanon.The SEEK trial was retrospectively registered at NIH reference number #NCT07008950, initially released on February 28th, 2025 and last released on June 5th 2025.

In this study, we report results from the baseline data collected before participant enrollment in the intervention. This study focusesspecifically on SRH-related characteristics of the recruited participants collected during November and December 2023, with a particular emphasis on STIs and HIV/AIDs.

SEEK was conducted at two Primary Healthcare Centers (PHCs) in the Beqaa governorate of Lebanon, which hosts the largest proportion of Syrian refugees in the country, accounting for approximately 39% [11]. These two PHCs were selected based on the study’s eligibility criteria.Participants were recruited through a coordinated process with the two selected PHCs. Using the PHCs’ patient databases, clinic staff identified eligible Syrian refugee adolescent girls and young women based on predefined inclusion criteria. Potential participants (or their legal guardians if under 18) were contacted via telephone and invited to the PHC to complete screening and participate in the baseline survey. Trained research assistants supported PHC staff with scheduling and on-site logistics. Recruitment continued until the target sample size was achieved. Participants were not randomly sampled, but were drawn from the pool of existing PHC users. All participants provided informed consent or assent, and were offered optional OBGYN consultations, hygiene kits, and transportation reimbursement as participation incentives. For AGYW aged 15–17 years, written informed assent was obtained from the adolescent, and written informed consent was obtained from both parents when available. In cases where both parents or one parent were absent, consent was obtained from two legally recognized guardians (if both parents were absent), or from one parent and one legal guardian (if only one parent was absent), in accordance with the study’s ethics approval.

Further details on the study’s target population, eligibility criteria, sampling strategy,, data collection tools and procedures are reported elsewhere (paper currently under review), however they are accessible as supplementary material attached within this manuscript.

Coincidentally, data collection was conducted at the onset of minor active conflict in the South of Lebanon before its escalation later that year. To a large extent this may not have had a severe impact on participation because during that time the conflict was still contained.

This study primarily reported findings on STIs, knowledge ofHIV/AIDS and its transmission methods, and health-seeking behaviors for STIs as key outcomes, while also examining influencing factors from other sections. In this study, the Pan Aarab Project for Family Health (PAPFAM) was used to collect SRH-related data with four variablesbeing considered outcomes of interest: Experience of any STI Symptoms (categorized into Yes and No), Healthcare-Seeking Behaviors (categorized into Yes and No); Previous knowledge ofHIV/AIDS (categorized into Yes and No), and Knowledge ofHIV/AIDS transmission methods (knows all, some, or none of the methods). The PAPFAM has been already used in several other studies and details regarding the tool have been published elsewhere [24–27].

Knowledge of HIV/AIDS transmission methods was assessed through different questions (e.g. unprotected sex, blood transfusion, etc…) with answers scored as 1 (knows the method) or 0 (does not know the method) as used in a previous study [24] Participants who knew more methods of transmission received a high score of knowledge.

### Statistical analysis

Data was collected and coded into an Excel sheet and exported for analysis using Stata SE version 18. Categorical data are presented using frequency and percent. Four variables were considered as outcomes of interest: Experience of any STI Symptoms (categorized into Yes and No), Healthcare-Seeking Behaviors (categorized into Yes and No); Previous knowledge ofHIV/AIDS (categorized into Yes and No), and Knowledge of HIV/AIDS transmission methods (knows all, some, or none of the methods). Bivariate analysis was used to explore the percentage of suboptimal outcomes vis-à-vis each of the independent variables. Independent variables were grouped by demographics. Differences in proportions were tested using Pearson’s chi-square. Three multivariable logistic regression models were built, one per outcome variable, using the stepwise method and including all independent variables with *p* ≤ 0.05 at bivariate analysis in the initial model. Results are reported as odds ratio (OR) and 95% confidence interval (CI). Variables with rare occurrences were excluded from multivariable models to avoid unstable estimates, even if statistically significant. Analyses were carried out at a significance level of 0.05.

#### Ethical considerations

Ethical approval was sought and obtained from the Institutional Review Board (IRB) at the American University of Beirut. In line with these ethical guidelines, adolescent girls and young women refugees who displayed symptoms of distress were provided with a referral list of Non-Governmental Organizations (NGOs) that offer free mental health services. Participants were also well informed about their roles and rights in the research especially with regards to confidentiality and privacy. They were informed that their names would not appear in any subsequent outputs associated with the research as results would be disseminated in aggregate. They were also informed that they had the right to refrain from answering any question, or to drop out from the study at any time and for any reason. All participants were required to sign a written informed consent form before participating in research activities. Participants under 18 were also required to have their parents sign a consent form for their participation.The team also made sure that all data collectors were recruited to be women to facilitate communication with participants and reduce potential boundaries associated with the sensitive nature of the topic. Participants were finally informed that their participation, or lack thereof would not in any way impact services they are otherwise entitled for positively or negatively.

## Results

### Socio-demographics characteristics

485 women were included in the analysis (see Table 1 Supplementary). Of the 485 participants, 99 women (20.4%) are less than 18 and 368 women (79.6%) are more than 18. The median spouse age was 25 years old with 45.2% of spouses are 25 or less while 54.8% are older. Table 2(see supplementary material) presents the experience, behavior, and knowledge related to STIs and AIDS specifically among the 485 participants. A significant proportion (84.9%) of the women reported experiencing at least one symptom associated with STIs. The most common symptoms included pain or a burning sensation when urinating (58.8%), For health-seeking behavior, 42.9% of the women sought professional consultation about the problem, while the Majority remaining 57.1% did not. Concerning family planning, 65.1% of the women reported having used family planning methods related to protected sex in the past. Regarding knowledge about AIDS was generally moderate, with 62.1% of the participants acknowledging awareness of the disease, although 37.9% did not hear of AIDS. In terms of knowledge about transmission methods, 52.2% of the participants were unaware of any methods of transmission.

This demographic data is reported from another paper that is currently under review [28]. It has been added here for additional context prior to reporting subsequent bivariate and multivariate analyses.

### Bivariate analyses

Table 1 highlights the association of 4 dependent variables (outcomes): Having any symptom of STI, Seeking professional health care, previous knowledge of AIDS disease, and knowledge of AIDS transmission methods.

**Table 1:** Bivariate association of experiencing STI symptoms, Health-Seeking behaviors and knowledge of AIDS and its transmission methods with sociodemographic characteristics (*N* = 485)

	Experienced any STI Symptoms			Seek Professional care			Previous Knowledge of HIV/AIDS			Knowledge of HIV/AIDS Transmission Methods				
	Yes			Yes			Yes			Some		All		
	*n*	(%)	Sig.	*n*	(%)	Sig.	*n*	(%)	Sig	*n*	(%)	*n*	(%)	Sig.
Women Age			**0.021**			0.214			0.290					0.498
* ≤ 18 Years*	88	(88.9)		37	(37.4)		66	(66.7)		35	(35.4)	17	(17.2)	
* >18 Years*	324	(83.9)		171	(44.3)		235	(60.9)		144	(29.5)	66	(17.1)	
Spouse Age			0.603			0.144			**< 0.001**					**< 0.001**
< 25 Years	184	(84.0)		86	(39.3)		102	(46.6)		54	(24.7)	23	(10.5)	
>25 Years	228	(85.7)		122	(45.9)		199	(74.8)		95	(35.7)	60	(22.6)	
Woman Education			**0.027**			0.079			**< 0.001**					**0.001**
* Illiterate*	44	(73.3)		18	(30.0)		29	(48.3)		16	(26.7)	4	(6.7)	
* Intermediate*	343	(86.6)		179	(45.2)		244	(61.6)		127	(32.1)	60	(15.2)	
* Secondary*	24	(85.7)		11	(39.3)		28	(100)		6	(21.4)	19	(67.9)	
Spouse Education			0.835			0.587			**0.044**					0.549
* Illiterate*	68	(83.9)		34	(41.9)		43	(53.1)		21	(25.9)	15	(18.5)	
* Intermediate*	274	(85.9)		138	(43.3)		203	(63.6)		103	(32.3)	55	(17.2)	
* Secondary*	36	(87.8)		21	(51.2)		31	(75.6)		14	(34.2)	10	(24.4)	
Woman Working Status			0.421			0.641			0.510					0.735
* Not Working*	346	(84.4)		174	(42.4)		257	(62.7)		127	(30.9)	72	(17.6)	
* Currently Working*	66	(88.0)		34	(45.3)		44	(58.7)		22	(17.6)	11	(14.7)	
Spouse Working Status			. 265			0.453			0.995					0.423
* Not Working*	79	(88.8)		35	(39.3)		55	(61.8)		24	(26.9)	13	(14.6)	
* Currently Working*	383	(84.1)		173	(43.7)		246	(62.1)		125	(31.6)	70	(17.7)	
Spouse’s Current Wife Count			**0.043**			0.258			0.132					**0.011**
* One Wife*	390	(84.2)		196	(42.3)		284	(61.3)		136	(29.4)	80	(17.3)	
* More than one wife*	22	(100)		12	(54.6)		17	(77.3)		13	(59.1)	3	(13.6)	
Family Planning			**0.046**			0.252			**0.008**					**0.001**
* No*	132	(80.9)		65	(39.9)		89	(54.6)		50	(30.7)	15	(9.2)	
* Yes*	167	(87.8)		138	(45.4)		204	(67.1)		96	(31.6)	67	(22.0)	
Barriers to Healthcare														
Financial Barrier			**0.004**			0.386			**0.034**					0.096
* No*	141	(78.8)		72	(40.2)		100	(55.9)		47	(26.3)	20	(15.1)	
* Yes*	270	(88.5)		135	(44.3)		200	(65.6)		102	(33.4)	55	(18.0)	
Transportation Barrier			0.256			0.695			0.053					0.299
* No*	83	(81.4)		42	(41.2)		55	(53.9)		28	(27.5)	14	(13.7)	
* Yes*	329	(85.9)		166	(43.3)		246	(64.2)		121	(31.6)	69	(18.0)	
Embarrassment Barrier			**0.049**			0.253			0.457					0.641
* No*	252	(81.8)		120	(38.9)		187	(60.7)		86	(27.9)	53	(17.2)	
* Yes*	27	(96.4)		14	(50.0)		199	(67.9)		10	(35.7)	5	(17.9)	

Bolds mean ‘statistically significant *p* value’

Women’s age was associated with experiencing STI symptoms, with younger women -under 18 years- reporting a higher prevalence of STI symptoms, compared to older women (*p-value* = 0.021). However, for their spouses, higher age was associated with a higher level of knowledge aboutHIV/AIDS and its transmission methods, with spouses older than 25 years having a higher percentage of knowledge aboutHIV/AIDS compared to those with younger spouses ((*p-value* < 0.001). Similarly, forHIV/AIDS transmission, the percentage of women who knew some or all methods ofHIV/AIDS transmission was higher among those with older spouses.

In terms of education, women with lower levels of education (illiterate) had the lowest percentages of seeking care, knowledge ofHIV/AIDS, and knowledge ofHIV/AIDS transmission, whereby only few participants reported that they know all methods compared to women with intermediate or secondary education. As for spouse education, it was only associated with knowledge of HIV/AIDS, whereby participants whose spouses had secondary level education had higher knowledge than those whose spouses were illiterate (p-value = 0.044).

Experiencing any STI symptoms and knowledge ofHIV/AIDS transmission were associated with having multiple wives. All of the women whose spouses had multiple wives experienced STIs **(***p-value* = 0.034). Also, the percentage of those who had some knowledge ofHIV/AIDS transmission methods was higher among those whose spouses have more than one wife (*p-value* = 0.011).

The barriers to treatment, including transportation and embarrassment had varied impacts, with embarrassment being linked to a higher likelihood of seeking professional care among those who felt embarrassed, but having little impact on overall knowledge. Women who reported having financial barriers to seeking healthcare also reported higher prevalence of STI symptoms (p-value = 0.004). However, women who faced financial barriers had higher knowledge ofHIV/AIDS than those who did not (p-value = 0.034). Women with better family planning access showed better knowledge ofHIV/AIDS transmission, with the Majority reporting some knowledge and 31.6% having more comprehensive knowledge, compared to those without access.

### Multivariable logistic regression

#### Experiencing STI symptoms

Table 2 presents the multivariate logistic regression models which included all variables with significant bivariate associations. Older age of women emerged as a significant predictor for experiencing STI symptoms, with those over the age of 18 having 42% lower odds of experiencing STI symptoms compared to younger women (aOR = 0.58, 95%CI= [0.25,0.84], p-value = 0.025). Reporting financial barriers to seeking healthcare was significantly associated with nearly twice the odds of experiencing STI symptoms (aOR = 1.99, 95% CI = [1.07, 3.69], p-value = 0.028). Additionally, the present use of family planning methods, mainly protected sex methods, was significantly associated with higher odds of experiencing STI symptoms (aOR = 1.88, 95%CI= [1.01,3.51], p-value = 0.045).

**Table 2:** Multivariable logistic regressions models of experiencing STI symptoms, knowledge of HIV/AIDS disease, and knowledge of HIV/AIDS transmission methods

	Experienced any of STI Symptoms			Previous Knowledge of HIV/AIDS			Knowledge of HIV/AIDS Transmission Methods					
	Yes, vs. No (ref)			Yes, vs. No (ref)			Some vs. None			All vs. None		
	aOR	[95% CI]	Sig.	aOR	[95% CI]	Sig.	aOR	[95% CI]	Sig.	aOR	[95% CI]	Sig.
Woman Age												
* > 18 years vs. ≤ 18 as ref*	0.58	[0.25,0.84]	**0.025**	0.17	[0.44;1.23]	0.255	0.69	[0.41,1.17	0.175	0.95	(0.47,1.90)	0.175
Spouse Age												
* > 25 years vs. ≤ 25as ref*	NA	NA	NA	3.087	[1.97;4.83]	**0.000**	2.20	[1.42,3.39	**0.000**	2.62	(1.46;4.70)	**0.001**
Wife Education												
* Intermediate vs. illiterate*	1.69	[0.70;4.06]	0.241	2.127	[1.08;4.11]	**0.028**	1.63	[0.83;3.17	0.150	2.74	(0.9;8.18)	0.071
* Secondary vs. illiterate*	0.97	[0.21;4.44]	0.968	**	**	**	4.54	1.00;21.22	**0.050**	3.99	(2.33;5.64)	**0.000**
Spouse Education												
* Intermediate vs. illiterate*	NA	NA	NA	1.192	[0.68;2.08]	0.537	NA	NA	NA	NA	NA	NA
* Secondary vs. illiterate*	NA	NA	NA	1.439	[0.56;3.69]	0.448	NA	NA	NA	NA	NA	NA
Spouse’s Current Wife Count												
* More than 1 wife (ref: 1 wife)*	**	**	**	NA	NA	NA	3.92	[1.41;10.84	**0.008**	1.76	[0.38;8.11]	0.465
Embarrassment												
* Yes*,* vs. No as ref*	4.80	[0.62;37.15]	0.132	NA	NA	NA	NA	NA	NA	NA	NA	NA
Financial barrier												
* Yes*,* vs. No as ref*	1.99	[1.07;3.69]	**0.028**	1.668	[1.06; 2.60]	**0.024**	NA	NA	NA	NA	NA	NA
Use FP												
* Yes*,* vs. No as ref*	1.88	[1.01;3.51]	**0.045**	1.604	[1.01;2.52]	**0.042**	1.28	(0.82;2.0]	0.273	3.21	[1.63;6.33]	**0.001**

NA: Not a candidate predictor to be considered in multivariate regression due to lack of significance in the bivariate analysis

*aOR* Adjusted OR

Bold means statistically significant result at p < 0.05

**Variable is omitted from the regression model due to the low cell count

#### Knowledge ofHIV/AIDS

Women whose spouses were older than 25 years had significantly higher odds of having previous knowledge ofHIV/AIDS (aOR = 3.08, 95%CI= [1.97, 4.83]). Wife’s education was also a significant factor, as those with intermediate education were twice as likely to have heard ofHIV/AIDS compared to illiterate women (aOR = 2.12, 95% CI = [1.08, 4.11], p-value = 0.028). Financial barriers to healthcare were significantly associated with previous knowledge of HIV/AIDS, with women facing such barriers being more likely to have prior knowledge (aOR = 1.668, 95% CI= [1.06–2.60], p-value = 0.024). Additionally, FP use was associated with increased odds of prior knowledge ofHIV/AIDS (aOR = 1.604, 95% CI= [1.01, 2.52], p-value = 0.042).

#### Knowledge ofHIV/AIDS transmission methods

Similar patterns were observed regarding knowledge ofHIV/AIDS transmission methods. Having a spouse older than 25 years was significantly associated with increased odds of knowing allHIV/AIDS transmission methods (aOR = 2.62, 95% CI=[1.46, 4.70], p-value = 0.001). Women’s education also played a significant role, with those having secondary education being nearly four times more likely to have full knowledge ofHIV/AIDS transmission as compared to illiterate women (aOR = 3.99, 95% CI = [2.33, 5.64], p-value = 0.000). Additionally, women whose spouses had multiple wives had a higher odds of knowing about some transmission methods of HIV/AIDS, showing a threefold increase in odds (aOR = 3.92, 95% CI = [1.41, 10.84], p-value = 0.008). Furthermore, use of family planning was strongly associated with knowledge ofHIV/AIDS transmission methods, with women using family planning methods having significantly higher odds of knowing all transmission methods (aOR = 3.21, 95% CI [1.63, 6.33], p-value = 0.001).

## Discussion

This study examined the associations between STI symptoms experienced, health-seeking behaviors, and knowledge ofHIV/AIDS with the sociodemographic and clinical correlates among Syrian adolescent girls and young women refugees in Lebanon. Given the limited data on this issue, these findings provide crucial insights into key associations, and highlight related vulnerabilities to inform research, practice, and policy aiming to improve outcomes, including STIs, healthcare access, and prevention among this population [12, 29]. The study population was characterized by limited education, low employment among women, and significant barriers to healthcare access, primarily due to financial constraints and transportation challenges.

Our findings indicate a high prevalence of STI symptoms experienced among participants (details reported in a separate manuscript currently under review), exceeding the reported rate of 50.2% among Syrian women who migrated to Turkey [20]. We found that women of higher age were significantly less likely to experience STI symptoms, consistent with previous research suggesting that adolescent Syrian refugee girls often face higher risks of poor SRH outcomes, including STIs, due to forced displacement, disruption of education and early marriage [6, 10, 12]. These vulnerabilities may also be influenced by religious contexts. In many conservative settings, particularly those shaped by religious institutions, young and unmarried women, may have limited access to comprehensive sexual and reproductive health (SRH) education, especially regarding safe sex and STI prevention. This lack of information may contribute to increased vulnerability and a higher prevalence of STIs among adolescent girls [30]. Financial barriers to accessing healthcare were significantly associated with a greater likelihood of experiencing STI symptoms, in line with the literature showing that extreme poverty and limited access to SRH services may increase STI vulnerability among Syrian refugee women in Lebanon [6, 29]. Additionally, our study found that FP use, particularly protected sex methods, was significantly associated with higher odds of experiencing STI symptoms. While uncommon in the literature, this finding may reflect the complex dynamics of FP use in refugee contexts. It may also indicate more frequent engagement with healthcare services among FP users, potentially resulting in greater reporting of STI symptoms, or it may also be due to our lack of measurement regarding the temporality of these variables. It is possible that those who had experienced STI symptoms resorted to using protected sex methods, however our study design may prohibit establishing the temporality of this association. Ultimately, this highlights the need for further investigation to explore the behavioral factors that may underlie this association. The study’s design, being focused on self-reports of symptoms as opposed to clinical assessments, may also result in underreporting or over-reporting, as this is a commonly found challenge in such self-reported survey designs [31].

Several socio-demographic variables predicted knowledge ofHIV/AIDS among study participants. Participants with higher education levels and whose spouses were older than 25 years were more likely to know about HIV/AIDS. However, no significant association was found with women’s age, partially contradicting existing literature, which shows a strong association between older age and higher education levels with HIV/AIDS knowledge among Syrian refugee girls in Lebanon [10]. This contradiction and lack of association may be explained by the relatively young age of women in our sample. Women facing financial barriers to healthcare were significantly more likely to have heard of HIV/AIDS, which contradicts literature findings suggesting that such barriers to accessing SRH services often limit knowledge on SRH issues [8]. This finding may reflect the unique dynamics in humanitarian contexts that warrant further investigation. Further longitudinal or qualitative research is needed to explore the relationship between financial constraints and exposure to SRH information within refugee contexts. Using FP methods was strongly associated with better knowledge of HIV/AIDS, possibly because women engaged in FP have greater knowledge and access to SRH services. This aligns with research showing that limited access to health services leads to poor SRH knowledge among migrant and refugee women [18].

Women with spouses older than 25 demonstrated better knowledge ofHIV/AIDS transmission methods, contradicting existing literature that found no significant association between husband’s age and refugee women’s knowledge of HIV transmission methods [32]. This may be explained by the possibility that older spouses have greater awareness of health issues, such as HIV/AIDS, and may share their knowledge with their wives. Additionally, women with higher education levels were more likely to know all transmission methods, similar to findings from a cross-sectional study on Rohingya refugee women in Bangladesh, where formal education was linked to better knowledge ofHIV/AIDS transmission methods [32]. Higher education may improve access to health information and enhance knowledge, however, given that most women in our sample had low education levels, overall awareness of transmission methods may have been limited. Women who used FP methods were significantly more aware of all transmission methods as well, which could be attributed to their increased access to healthcare services. This aligns with existing literature where availability of healthcare services was associated with better knowledge ofHIV/AIDS transmission among refugee women [32].

### Implications

This study highlights several modifiable factors that may inform targeted SRH interventions for Syrian refugee adolescent girls and young women in Lebanon. Most importantly, given our finding that financial barriers predict poorer STI outcomes it is important to reduce financial barriers to healthcare through subsidized SRH service packages, reimbursing transportation costs, or integrating low-cost STI screening within existing FP visits. Similarly, structured SRH education modules may be embedded into routing FP consultations at the PHCs in a manner that is tailored to adolescents and young women. As for safe sexual behavior, it may be important to link awareness sessions with FP consultations not only for women and adolescent girls, but also for male partners to improve negotiation and attitudes towards FP use. In this regard, it may be important to incorporate language and messages that are sensitive to religious beliefs to improve support for FP uptake. Future research may also consider the inclusion of validated measures of religiosity such as frequency of prayer or adherence to gender norms, to better understand how religious observance may influence SRH knowledge, attitudes, and behaviors in refugee settings.

### Limitations

This study includes several limitations. First, thestudy design, reporting baseline data from an RCT, limits the ability to establish causal relationships between the identified factors and outcomes. Second, reliance on self-reported data, as opposed to medical and clinical assessments, on sensitive topics, including STIs and HIV/AIDS, may introduce social desirability and recall biases potentially due to stigma or misinterpretation of symptoms, impacting data precision and the accuracy of reported information as seen with some findings that contradict previous literature. Also, some underreporting or misreporting may still have occurred due to stigma surrounding these topics. In addition, the operational definition of “STI symptoms” relied on self-reported experiences of non-specific urogenital complaints (e.g., itching, discharge, dysuria), which may stem from both infectious and non-infectious causes [33–35]. This likely overestimates the true prevalence of clinically confirmed STIs and may blur the distinction between general urogenital discomfort and actual infections. The lack of clinical or microbiological confirmation is a limitation inherent to self-report-based studies in low-resource settings. However, this limitation is partially addressed by the fact that we used the PAPFAM tool which is well established for measuring such indicators. Furthermore, the study was conducted only in two primary healthcare centers in the Bekaa governorate in Lebanon. As the sample was drawn exclusively from existing PHC users, it may not fully represent the broader refugee population particularly, those who do not seek formal healthcare services. This potential selection bias may compromise external validity and limits the ability to extrapolate these findings to other settings. That said, this risk is minimal because PHCs tend to offer free-of-charge services to refugee populations and identifying participants from PHCs may therefore cover a large portion of Syrian refugee populations within their catchment areas. Finally, the lack of qualitative data limits the ability to capture nuanced perspectives and contextual factors that might influence the reported outcomes. Future research, including a more representative sample and incorporating qualitative components, is needed to better understand STI correlates among refugee populations. In particular, the study did not include measures of religious affiliation or level of observance, which may be important socio-cultural variables influencing SRH knowledge and behaviors.

## Conclusion

This study examined sociodemographic and clinical associations with STI symptoms, health-seeking behaviors, and knowledge ofHIV/AIDS among Syrian refugee adolescent girls and young women in Lebanon. This study highlights several important associations. For instance, higher age of women is associated with less experiences of STI symptoms, whereas use of FP methods and financial barriers are associated with poorer STI symptoms. Similarly, higher age of spouse, higher education among participants, and use of FP methods were associated with better knowledge ofHIV/AIDS and its transmission methods.

These findings have several policy implications, including a call for strengthening the integration of STI prevention and education into family planning and PHC services tailored for Syrian refugee women and girls. Policy makers are also recommended to address financial and structural barriers to healthcare through subsidized services and transportation support, with particular attention to younger and less educated refugee women who may face compounded vulnerabilities.

## Supplementary Information

Supplementary Material 1.

## Data Availability

The datasets used and/or analyzed during the current study are available from the corresponding author on reasonable request.

## Abbreviations

AIDS: Acquired immune deficiency syndrome
ELRHA: Enhancing learning and research in humanitarian assistance
FP: Family planning
NGO: Non-governmental organization
PHC: Primary healthcare center
RCT: Randomized controlled trial
STI: Sexually transmitted infection
SRH: Sexual reproductive health
WHO: World health organization
PAPFAM: Pan arab project for family health
IRB: Institutional review board
OR: Odds ratio
aOR: Adjusted odds ratio
