# Supplementary material for: Correlates of sexually transmitted infections among Syrian refugee women and girls in Lebanon: knowledge, symptoms, and health-seeking behaviors
Source: BMC Womens Health. 2025 Oct 8;25:477. doi: 10.1186/s12905-025-04036-z (PMC12506282; doi:10.1186/s12905-025-04036-z)
Supplement: Supplementary file 1 — Supplementary Material 1. [file 12905_2025_4036_MOESM1_ESM.docx]

Supplementary Material (taken from Naal et al. (39).

Methodology:

The sample included Syrian refugee women and girls aged 15-24, living in the Beqaa region in Lebanon and who normally attend one of the two selected PHCs. To be eligible for the study, participants had to be married, not breastfeeding, not pregnant, not suffering from a chronic disease, and willing to commit to enrollment in the study.

Participants were recruited from two PHCs recommended by the Ministry of Public Health in Lebanon. PHCs were first requested to identify potentially eligible candidates from their databases, based on their records. A research assistant contacted these individuals by phone using an invitation script, and invited them to participate in baseline data collection between November and December 2023. The research assistant reached out directly to participants over 18. As for participants under 18, their legal guardians were contacted.

The sample size was determined to be 380 adolescent girls and young women because it assumed, based on reported data, a baseline prevalence of 30% family planning use in the sample, and so this sample size would provide sufficient power to detect significant changes by 20%. To that end, a total of 536 participants were invited to participate in the screening and baseline data collection, of which 485 were found to be eligible and subsequently completed the baseline questionnaire. Data reported in this study represents the full initial sample prior to sub-sampling and randomization.

| **Table 1. Participant Sociodemographic Characteristics (n=485)** | | | | |
| --- | --- | --- | --- | --- |
| **n** | | | | **(%)** |
| **Age** | | |  |  |
|  | | **Wife** |  |  |
|  | | *< 18 years* | 99 | (20.4) |
|  | | *> 18 years* | 368 | (79.6) |
|  | | **Spouse** |  |  |
|  | | *< 25 years* | 219 | (45.2) |
|  | | *> 25 years* | 266 | (54.8) |
| **Education** | | |  |  |
|  | **Wife** | |  |  |
|  | *Illiterate* | | 60 | (12.4) |
|  | *Intermediate and below* | | 396 | (81.8) |
|  | *Secondary and above* | | 28 | (5.8) |
|  | **Spouse** | |  |  |
|  | *Illiterate* | | 81 | (18.7) |
|  | *Intermediate and below* | | 319 | (72.3) |
|  | *Secondary and above* | | 41 | (9.3) |
| **Working Status** | | |  |  |
|  | **Wife** | |  |  |
|  | *Currently Working (Yes)* | | 75 | (15.5) |
|  | **Spouse** | |  |  |
|  | *Currently Working (Yes)* | | 369 | (76.1) |
| **Spouse’s Current Wife Count** | | |  |  |
|  | | *One wife* | 463 | (95.5) |
|  | | *More than one wife* | 22 | (4.5) |
| **Kinship with the Spouse** *(Yes)* | | | 248 | (51.1) |
| **Barriers to Seek Healthcare** | | |  |  |
|  | **Financial Barrier** *(Yes)* | | 305 | (63.1) |
|  | **Transportation Barrier** *(Yes)* | | 295 | (60.8) |
|  | **Feeling Embarrassed to Seek Care** *(Yes)* | | 38 | (8.3) |
|  |  |  |  |  |

| **Table2. Experience of Sexually Transmitted Infections Symptoms, Health-Seeking Behaviors, and Knowledge of HIV/AIDS**  **and its Transmission Methods (n=485)** | | | | | | |
| --- | --- | --- | --- | --- | --- | --- |
|  | |  | **n** | | **(%)** | |
| **Experience of any Sexually Transmitted Infection Symptoms (Symptoms)** | |  | 412 | (84.9) | | |
|  | *Pain or burning sensation when urinating (yes)* |  | 285 | (58.8) | | |
|  | *Sharp or unfamiliar vaginal discharge (yes)* |  | 294 | (60.6) | | |
|  | *Itching or allergy around the reproductive system (yes)* |  | 249 | (51.3) | | |
|  | *Foul-smelling discharge(yes)* |  | 241 | (49.9) | | |
|  | *Sharp pain in the lower abdomen unrelated to the menstrual cycle(yes)* |  | 284 | (58.6) | | |
| **The woman sought professional ^a^ consultation about the problem *(Health-Seeking Behavior)*** | |  |  |  | | |
|  | *No* |  | 277 | (57.1) | | |
|  | *Yes* |  | 208 | (42.9) | | |
| **Use of Protected Sex as a Family Planning Method** | |  |  |  | | |
|  | *No* |  | 163 | (34.9) | | |
|  | *Yes* |  | 304 | (65.1) | | |
| **Knowledge of HIV/AIDS** | |  |  |  | | |
|  | *No* |  | 184 | (37.9) | | |
|  | *Yes* |  | 301 | (62.1) | | |
| **Knowledge of HIV/AIDS Transmission ^b^** | |  |  |  | | |
|  | *Doesn’t know any method* |  | 253 | | | (52.2) |
|  | *Know some of the methods of transmission* |  | 149 | | | (30.7) |
|  | *Know all of the methods of transmission* |  | 83 | | | (17.1) |
| a. Professional consultation includes consultations with healthcare providers only  b. Transmission methods include unprotected sex, drug injection, and blood transfusion | | | | | | |
|  |  |  |  |  |  |  |
